# Thrombospondin 1 in hypoxia-conditioned media blocks the growth of human microvascular endothelial cells and is increased in systemic sclerosis tissues

**DOI:** 10.1186/1755-1536-4-13

**Published:** 2011-06-02

**Authors:** Luke Morgan-Rowe, Joanna Nikitorowicz, Xu Shiwen, Andrew Leask, Janice Tsui, David Abraham, Richard Stratton

**Affiliations:** 1 Centre for Rheumatology Research and Connective Tissue Diseases, The Royal Free Hospital Campus, University College London, Pond Street, London NW3 2QG, UK

## Abstract

**Background:**

Systemic sclerosis (SSc) is a chronic inflammatory autoimmune disease characterised by vascular dysfunction and damage, excess collagen deposition and subsequent organ manifestations. Vasculopathy is an early feature of the disease which leads to a chronic hypoxic environment in the tissues. Paradoxically, there is a lack of angiogenesis. We hypothesised that this may in part be due to a nonphysiological, overriding upregulation in antiangiogenic factors produced by the hypoxic tissues. We considered thrombospondin 1 (TSP-1) as a candidate antiangiogenic factor.

**Results:**

Conditioned media from human microvascular endothelial cells cultured in both normoxic and hypoxic environments were able to block endothelial cell proliferation, with the latter environment having a more profound effect. Filtration to remove > 100-kDa proteins or heparin-binding proteins from the conditioned media eliminated their antiproliferative effect. TSP-1 was expressed in high concentrations in the hypoxic media, as was vascular endothelial growth factor (VEGF). Depletion of TSP-1 from the media by immunoprecipitation reduced the antiproliferative effect. We then show that, in a dose-dependent fashion, recombinant TSP-1 blocks the proliferation of endothelial cells. Immunohistochemistry of skin biopsy material revealed that TSP-1 expression was significantly higher throughout the skin of patients with SSc compared with healthy controls.

**Conclusions:**

Despite the environment of chronic tissue hypoxia in SSc, there is a paradoxical absence of angiogenesis. This is thought to be due in part to aberrant expression of antiangiogenic factors, including TSP-1. We have demonstrated that TSP-1 is released in high concentrations by hypoxic endothelial cells. The conditioned media from these cells is able to block proliferation and induce apoptosis in microvascular endothelial cells, an effect that is reduced when TSP-1 is immunoprecipitated out. Further, we have shown that recombinant TSP-1 is able to block proliferation and induce apoptosis at concentrations consistent with those found in the plasma of patients with SSc and that its effect occurs in the presence of elevated VEGF levels. Taken together, these data are consistent with a model wherein injured microvascular cells in SSc fail to repair because of dysregulated induction of TSP-1 in the hypoxic tissues.

## Background

Systemic sclerosis (SSc) is a chronic inflammatory autoimmune disease characterised by vascular dysfunction and damage, excess collagen deposition and subsequent organ manifestations [1]. The pathogenesis of SSc has yet to be fully elucidated. Vasculopathy occurs early in the disease and precedes fibrosis [2]. This is characterised by both abnormal vascular tone and endothelial cell damage [3]. Endothelial apoptosis is seen in recent-onset SSc patients' dermal biopsies [4]. Anti-endothelial cell antibodies have been detected in some but not all patients with SSc [5]. These antibodies are capable of upregulating the expression of endothelial cell adhesion molecules and inducing apoptosis [6].

Other sources of early endothelial cell damage include reactive oxygen species, markers of which are found in the serum and urine of patients with SSc at higher levels than in controls [7,8]. In addition, nitric oxide synthesis by endothelial cells is dysregulated in SSc because of suppression of the endogenous nitric oxide synthetase and upregulation of the inducible isoform. Because of this, proteins and lipids can become damaged in SSc by oxidation or nitrosylation [9].

Vascular damage clinically manifests in early SSc as Raynaud's phenomenon, sometimes seen many years before the other features develop [10]. In addition, nailfold capillaroscopy is abnormal in early SSc, reflecting the endothelial vascular damage. The characteristic features of nailfold capillaroscopy in SSc include the presence of abnormal tortuous microvascular loops, the progressive loss of capillary density and areas of microvascular loss. Despite the chronic hypoxic environment and increased levels of proangiogenic vascular endothelial growth factor (VEGF) and its receptors, there is a failure of endothelial repair and an absence of angiogenesis in SSc [11,12].

It has been postulated that the failure of angiogenesis in SSc may be due to a nonphysiological, overriding influence of antiangiogenic factors, which are present in high concentrations in the sera of patients with SSc [13-15]. Furthermore, plasma from patients with SSc has been shown to inhibit the migration and proliferation of microvascular endothelial cells [16].

Because of this, we became interested in the idea that hypoxic tissues are capable of releasing factors which interfere with reparative angiogenesis. We have gone on to test whether media from human cells cultured under hypoxic conditions can interfere with the growth of endothelial cells and whether the candidate antiangiogenic factor thrombospondin 1 (TSP-1) is induced in hypoxic cells or in the involved tissues of SSc patients.

## Methods

### Cell culture

The extended lifespan simian virus 40 transfected dermal human microvascular endothelial cell 1 (HMEC-1) cell line was employed. This line was generated by Ades *et al*. [17] and has been shown to maintain the phenotypic characteristics of small-vessel endothelial cells. For experiments, HMEC-1 cells were cultured in DMEM (Autogen Bioclear UK Ltd, Caine, Wiltshire, UK) with 10% FCS, 2 mM L-glutamine, 1 mM sodium pyruvate, 100 U/mL penicillin and 100 μg/mL streptomycin.

Cells were examined regularly by using phase contrast microscopy. Preparations which had reached confluence were trypsinised for secondary culture. Preparations which appeared to be infected by bacteria or failed to reach confluence after five days were discarded. Cells were passaged at confluence using 10% trypsin and split into equal thirds. The C_2_C_12 _mouse myoblast line 19 was also used. C_2_C_12 _cells were cultured on 0.01% gelatine plates in DMEM supplemented with 10% FCS, 2 mM L-glutamine, 1 mM sodium pyruvate, 100 U/mL penicillin and 100 μg/mL streptomycin.

### Hypoxic culture

Cells were cultured in a custom-made hypoxic chamber placed within a tissue culture incubator and maintained at 37°C. The cells were plated onto 100-mm tissue culture plates and placed within the chamber, which had been primed for one hour by flushing with 4% CO_2_, 1% O_2 _and 95% N_2_. The chamber was then sealed, and hypoxic conditions were maintained for a further 24 hours prior to removal of the media for assay. Media were removed at the end of 24 hours of hypoxic culture for gas analysis and were found to have the following values (means ± SEM): pH 7.41 ± 0.04, pO_2 _6.4 ± 0.8 kPa and pCO_2 _5.2 ± 1.2 kPa. By comparison, media following culture under standard normoxic conditions for 24 hours had the following values (means ± SEM): pH 7.42 ± 0.04, pO_2 _16.2 ± 1.1 kPa and pCO_2 _4.8 ± 1.6 kPa.

### Endothelial cell proliferation

HMEC-1 cells were harvested at 80% confluence and plated at 5 × 10^4 ^cells/well in 12-well plates, cultured for 24 hours and then cultured further in the presence or absence of 10% FCS with or without conditioned media from endothelial cells or skeletal muscle cells to test for antiproliferative effects of the conditioned media. After 24 hours of incubation, the cells were harvested by trypsinisation and counted in a haemocytometer. Each experiment was performed in triplicate.

In further experiments, HMEC-1 cells were cultured on 96-well plates with or without conditioned media from endothelial cells or with various concentrations of recombinant TSP-1 (R&D Systems Minneapolis, MN, USA). The number of viable cells was measured by WST-1 assay. In this assay, a tetrazolium dye undergoes a colour change depending on the presence of viable mitochondria. Ten microlitres of WST-1 were added to each well, and the cells were incubated for a further two hours. The colour intensity was read at 450 nm (reference wavelength 655 nm) in an ELISA plate reader.

### Assay for TSP-1 and VEGF

Media were removed and assayed for TSP-1 and VEGF by ELISA (R&D Systems Pharmacia Biotech, Little Chalfont, UK). Experiments were performed in triplicate, and samples were assayed in duplicate according to the manufacturer's instructions. Standard curves were included in each assay plate.

### Western blot analysis

Western lysate samples of 20 μL containing 10 μg of protein were run upon a ready-cast 4% to 12% Tris-Glycine gel (Novex. Ontario, Canada) alongside a broad-range protein marker (New England Biotech, Wakefield, MA, USA) at 125 V until the dye front had reached the bottom of the gel (approximately 1.5 hours) in Tris-Glycine Running Buffer (Invitrogen, Carlsbad, CA, USA). Proteins were electrophoretically transferred to nitrocellulose Hybond-C (Amersham Pharmacia, Little ChalfontBuckinghamshire, UK). Each membrane was briefly washed, and then nonspecific protein binding was blocked by one-hour incubation with 10% milk protein in PBS. Membranes were probed with antibodies against caspase 3 and cleaved caspase 3 (#9662 and #9661S; New England Biolabs, Wakefield, MA, USA), and binding was detected using labelled species-specific secondary antibody followed by avidin-biotin complex detection assay (Amersham Pharmacia).

### Quantitative RT-PCR analysis

HMEC-1 were cultured to 90% confluence and incubated overnight in hypoxic (1% O_2 _and 5% CO_2_) or normal conditions at 37°C. RNA was extracted from cells using the RNeasy Kit (Qiagen, Crawley West Sussex, UK), and the quality was evaluated on the Agilent 2100 Bioanalyzer (Agilent Technologies Edinburgh, UK). One microgram of RNA extracted from each well was reverse-transcribed using the QuantiTect Reverse Transcription Kit (Qiagen). qPCR was performed in triplicate using the SensiMix SYBR kit (Bioline, London, UK) on the Rotor-Gene 6000 (Qiagen, Crawley West Sussex, UK), and data were normalised against expression of *TUBB *and *SDHA*. The reference genes were chosen using genorm^PLUS ^software (Biogazelle, Ghent, Netherlands). The qRT-PCR conditions were 40 cycles at 95°C for 10 minutes, 95°C for 10 seconds, 60°C for 15 seconds and 72°C for 20 seconds. The primers used are shown in Table 1.

**Table 1:** Primers used for qRT-PCR

Gene	Primer	Sequence
THBS1	Forward Reverse	CTGACCTGAAATACGAATGTAGAGA TTTCTCAAGCCCATAGTTCCAGAAG
TUBB	Forward Reverse	ACATACCTTGAGGCGAGCAA TCACTGATCACCTCCCAGAA
SDHA	Forward Reverse	AGAAGCCCTTTGAGGAGCA CGATCACGGGTCTATATTCCAGA

### Immunohistochemistry

For immunofluorescence, sections were permeabilised by washing three times in cold PBS containing 0.3% Triton X-100 at room temperature for 5 minutes, then washing them further in PBS containing 0.1% Tween. Nonspecific binding sides were blocked with 3% BSA and 10% serum in PBS with Tween for 30 minutes. Sections were immunostained with rabbit polyclonal anti-TSP-1 antibody (#sc-14013; Santa Cruz Biotechnology, Santa Cruz, CA, USA) overnight at 4°C. As a negative control, samples were incubated with polyclonal rabbit immunoglobulin G (Vector Laboratories, Burlingame, CA, USA). Sections were incubated with Alexa Fluor 568- or Alexa Fluor 594-labelled anti-rabbit antibody (Molecular Probes/Invitrogen, Eugene, OR, USA) (1:1000) for one hour. Nuclei were stained for 4',6-diamidino-2-phenylindole (Sigma-Aldrich, St Louis, MO, USA) for 10 minutes. Glass coverslips were mounted onto slides with Vector aqueous anti-fade VECTASHIELD fluorescence mounting medium (Vector Laboratories) and sealed with nail polish. Sections were imaged with an Zeiss Axioplan microscope (Carl Zeiss, Heidenheim, Germany) at ×40 original magnification.

## Results

### Conditioned media from hypoxic endothelial cells block proliferation

**Figure 1:**
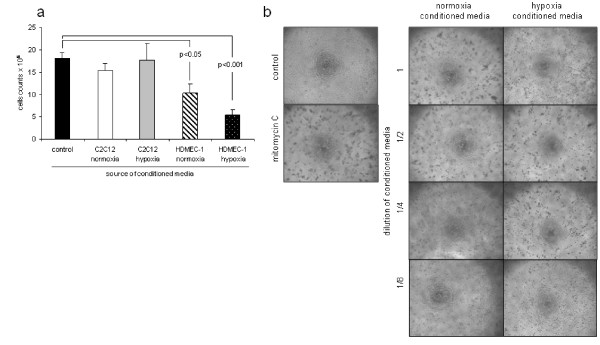
**Conditioned media from hypoxic endothelial cells inhibit proliferation**. Human microvascular endothelial cell 1 (HMEC-1) cells and C_2_C_12 _muscle cells were grown to confluence and then cultured further under normoxic or hypoxic conditions. Media were transferred undiluted to rapidly proliferating HMEC-1 cells in the presence of 10% FCS. Culture in DMEM with 10% FCS was used as a positive control, and culture with 10 μM mitomycin C added at baseline to block proliferation was used as a negative control. After 24 hours, further culture proliferation of HMEC-1 cells was measured using a haemocytometer, and the cells were imaged. **(a) **Exposure to conditioned media from HMEC-1 cells, but not from C_2_C_12 _cells, inhibited proliferation (results are expressed as total number of cells per well of a six-well plate/10^-4^). **(b) **Effects were more marked with media from hypoxic HMEC-1 cells which abolished proliferation and caused rounding up of the cells.

We began by testing whether media from hypoxic cells could block cellular proliferation. HMEC-1 endothelial cells and C_2_C_12 _muscle cells were cultured to confluence in 75-cm^2 ^flasks under normoxic or hypoxic conditions. After 24 hours, media were removed and transferred to proliferating HMEC-1 cells grown on six-well plates in 10% FCS. Culture under serum-free conditions was used as a negative control, and medium plus 10% FCS was used as a positive control. Conditioned media from HMEC-1 but not from C_2_C_12 _cells inhibited cellular proliferation. This effect was more marked when conditioned media from hypoxic HMEC-1 cells were used, and these were found to abolish proliferation (Figure 1). In addition, cells cultured in media transferred from hypoxic HMEC-1 cells took on an altered morphology with more rounding up of cells (Figure 1).

### Antiproliferative effect of hypoxia-conditioned media is due to a heparin-binding macromolecule

**Figure 2:**
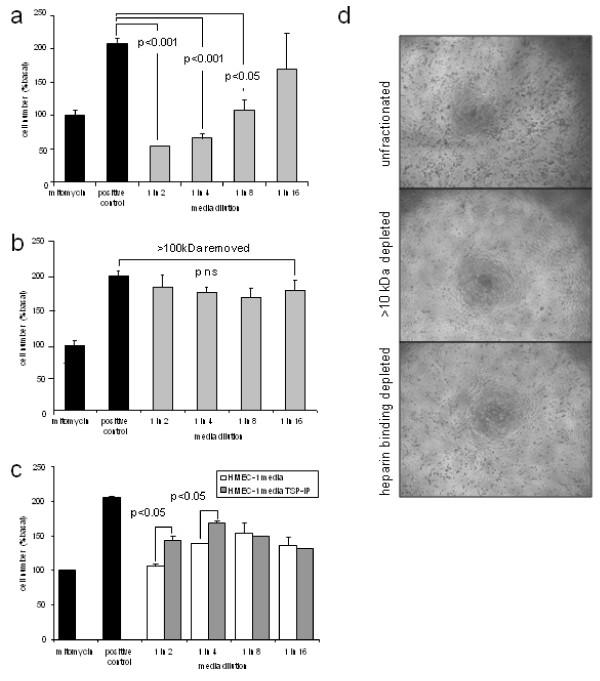
**Suppression of proliferation by endothelial cell-conditioned media is due in part to presence of heparin-binding macromolecule**. **(a) **In further experiments, HMEC-1 were cultured on 96-well plates in the presence of 10% FCS to promote proliferation. Treatment with mitomycin C at baseline was used as a negative control. Addition of conditioned media from hypoxic HMEC-1 blocked proliferation. **(b) **Filtration to remove molecules > 10 kDa, or heparin binding molecules blocked the effect of hypoxia conditioned media on morphology of treated cells. **(c) **In further experiments, filtration was used to remove molecules > 100 kDa or **(d) **immunoprecipitation of TSP-1 antagonised the antiproliferative effects of the conditioned media.

Because of the above-described findings, we became interested in identifying which factor released by hypoxic HMEC-1 cells was responsible for the suppression of cell proliferation and for the change in cellular morphology. Further experiments were performed using filtration to remove protein factors > 10 kDa and > 100 kDa and using heparin Sepharose beads to precipitate and remove heparin-binding factors. Media were transferred to proliferating HMEC-1 cells grown on 96-well plates, and cell numbers were assayed after 24 hours by WST-1 assay and imaged using a Zeiss Axio Scope (Carl Zeiss). Filtration to remove > 10-kDa factors was found to eliminate the antiproliferative effect of hypoxia-conditioned media and to block the morphological changes induced (Figure 2). Because of this, we concluded that the effects were not simply due to degradation of the hypoxia-conditioned media. Filtration of > 100-kDa factors was also found to abolish the antiproliferative effects of hypoxia-conditioned media (Figure 2). In addition, removal of heparin-binding proteins using heparin Sepharose beads led to loss of the antiproliferative effect of the media (Figure 2). We concluded that the antiproliferative effect of the hypoxia-conditioned media was due to the presence of heparin-binding macromolecules > 100 kDa.

### TSP-1 is induced in hypoxia-conditioned media and blocks proliferation of HMEC-1 cells

**Figure 3:**
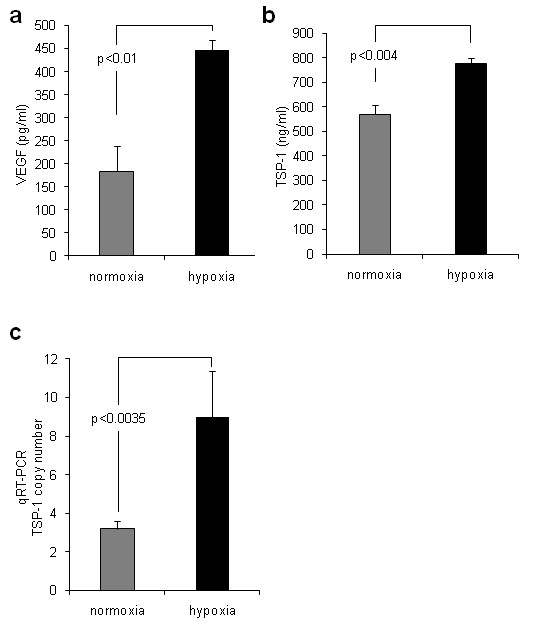
**Induction of VEGF and TSP-1 in the media of HMEC-1 cells cultured under hypoxic conditions**. Conditioned media from confluent HMEC-1 grown under normoxic or hypoxic culture conditions were assayed by ELISA for VEGF and TSP-1. Hypoxic culture (24 hours) was associated with increased levels of both **(a) **VEGF (mean ± SEM VEGF in normoxic conditions 184 ± 54 pg/mL, mean ± SEM VEGF in hypoxic conditions 447 ± 22 pg/mL; *P *< 0.05) and **(b) **TSP-1 (mean ± SEM TSP-1 in normoxic conditions 569 ± 37 pg/mL, mean ± SEM TSP-1 in hypoxic conditions 775 ± 24.5 pg/mL; *P *< 0.003). **(c) **In addition, TSP-1 mRNA levels were assayed by performing qRT-PCR. Hypoxic culture led to increased TSP-1 mRNA levels (mean ± SEM TSP-1 copy number in normoxia 3.19 ± 0.41, mean ± SEM TSP-1 copy number in hypoxia 8.98 ± 2.39; *P *< 0.004).

Because we found that the antiproliferative effects were due to the presence of a large heparin-binding factor, and because TSP-1 is believed to promote endothelial cell apoptosis, we considered TSP-1 as a candidate factor responsible for the antiproliferative effect and change in cellular morphology induced by hypoxia-conditioned media. We assayed levels of TSP-1 in media from HMEC-1 cells grown under normoxic and hypoxic conditions. TSP-1 was present in the media of normoxic HMEC-1 cells and was induced following 24 hours in hypoxic culture (mean ± SEM TSP-1 in normoxic HMEC-1 media 569 ± 37 ng/mL, mean ± SEM TSP-1 in hypoxic HMEC-1 media 775 ± 25 ng/mL; *P *< 0.004) (Figure 3). As expected, the hypoxic culture conditions led to an induction of VEGF, and levels of VEGF were increased in the hypoxic HMEC-1 media (mean ± SEM VEGF in normoxic HMEC-1 media 184 ± 54 pg/mL, mean ± SEM VEGF in hypoxic HMEC-1 media 447 ± 22 pg/mL, SEM; *P *< 0.01) (Figure 3).

In addition, TSP-1 mRNA levels in HMEC-1 cells cultured under normoxic and hypoxic conditions were assayed by qRT-PCR. Culture under hypoxic conditions for 24 hours led to the induction of TSP-1 expression (mean ± SEM normoxic cell TSP-1 copy number 3.19 ± 0.41, mean ± SEM hypoxic culture copy number 8.98 ± 2.39; *P *< 0.0035) (Figure 3).

**Figure 4:**
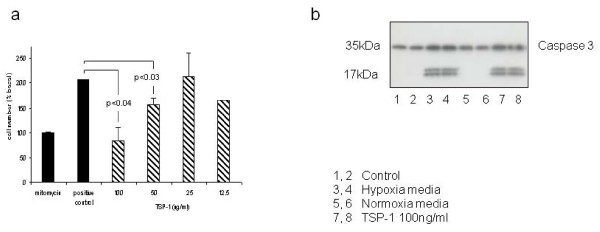
**Addition of recombinant TSP-1 to proliferating endothelial cells blocks proliferation and induces apoptosis**. **(a) **Recombinant human TSP-1 was added to rapidly proliferating HMEC-1 cells grown on 96-well plates in the presence of 10% FCS, and cell numbers were assessed by performing a WST-1 assay after 24 hours of further culture. The addition of TSP-1 suppressed proliferation in a dose-dependent manner. **(b) **In other experiments, proliferating HMEC-1 cells were treated with TSP-1 or conditioned media from cultured HMEC-1 cells and then lysed after 16 hours for Western blot analysis for cleaved caspase 3. Exposure to both TSP-1 and hypoxia-conditioned media led to elevation of cleaved caspase 3 in the cells, consistent with the induction of apoptosis. *P < 0.04. **P < 0.03.

Next we measured whether recombinant TSP-1 is able to block the proliferation of HMEC-1 cells. TSP-1 blocked, in a dose-dependent fashion, the proliferation of HMEC-1 cells grown on 96-well plates in the presence of 10% FCS (Figure 4). Full suppression of proliferation was seen with 100 ng/mL TSP-1 and partial suppression was observed with 50 ng/mL TSP-1. In addition, immunoprecipitation of TSP-1 from conditioned media blocked some but not all of the antiproliferative effect (Figure 2). Because we suspected that TSP-1 and hypoxia-conditioned media were causing apoptosis, we measured the effect of TSP-1 and hypoxia-conditioned media on cleaved caspase 3 levels in HMEC-1 cells. Both TSP-1 and hypoxia-conditioned media led to increased levels of cleaved caspase 3 in HMEC-1 cells after 16 hours of culture (Figure 4). We concluded that TSP-1 is induced following culture of HMEC-1 cells in hypoxia and that TSP-1 blocks the proliferation of HMEC-1 cells and causes apoptosis, despite the elevation of VEGF.

### TSP-1 is expressed by endothelial cells in normal skin and is induced in SSc-involved tissues

**Figure 5:**
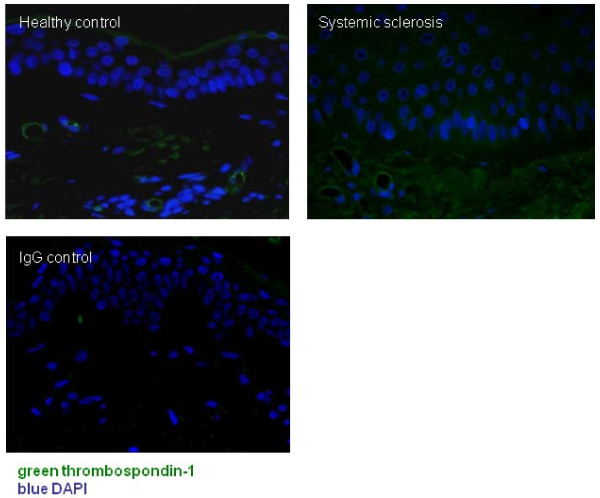
**TSP-1 is increased in the involved forearm skin of SSc patients**. Skin biopsies from SSc patients and healthy controls (*n *= 5 for each group) were stained by immunohistochemistry using a primary antibody against TSP-1. In healthy controls, positive staining was seen around endothelial cells in the dermis. In SSc sections, TSP-1 was increased and expressed in endothelial cells, epidermal keratinocytes and cells of the dermis. Isotype-matched antirabbit IgG polyclonal antibody was included as a control.

Because of the above-described findings, we became interested in the idea that similar effects might be occurring in SSc patients and that TSP-1 might be induced in the ischaemia-involved SSc tissues. We next performed immunohistochemistry to stain healthy control skin biopsy material and SSc-involved forearm skin for the presence of TSP-1 using a monoclonal anti-TSP-1 antibody. In healthy control skin, positive staining for TSP-1 was seen in the dermal microvasculature but was otherwise absent. By contrast, in SSc-involved skin, TSP-1-positive staining was observed in endothelial cells, epidermal keratinocytes and dermal connective tissue cells (Figure 5). We concluded that TSP-1 was being induced in the SSc dermis and epidermis.

Also, we wanted to test whether free TSP-1 was present in the dermal interstitial fluid sampled from SSc and healthy control skin. In fact, in dermal interstitial fluids, TSP-1 was present only at very low levels and appeared absent from SSc dermal fluid (data not shown). We concluded that while TSP-1 was abundant within the dermis and epidermis in SSc, it was present only at low levels as a free, non-matrix-associated factor in the extracellular fluid.

## Discussion

Hallmarks of SSc are tissue ischaemia due to vascular injury, remodelling and stenosis; increased vascular tone; and microvascular damage. Despite the endothelial cell damage and activation and the ensuing chronic hypoxic environment, there is a failure of angiogenesis in SSc. It has been hypothesised that this might be due in part to an aberrant expression of antiangiogenic factors, and we have sought to investigate further the antiangiogenic effects of TSP-1 in the disease.

TSP-1 is derived from a large trimeric glycoprotein and functions as an adhesive matricellular factor modifying the binding of a variety of cells to extracellular matrix proteins [18,19]. TSP-1 has multiple binding sites, including an amino terminal heparin-binding domain, a procollagen domain, epidermal growth factor-like repeats and an RGD (arginine-glycine-aspartic acid) integrin-binding region [20]. The binding of TSP-1 to fibronectin, fibrinogen, laminin and collagen 5 has been described [21,22]. TSP-1 is released from granules of platelets, from endothelial cells and from other cells, including keratinocytes and fibroblasts [23,24]. Diverse functions of TSP-1 have been defined, including the adhesion of platelets to thrombin-containing clots, targeting of cells for apoptosis and clearance of apoptotic cells by macrophages [25]. Studies of TSP-1-knockout mice have demonstrated that wounds heal more slowly and irregularly because of prolonged inflammation, delayed closure, scab loss and reepithelialisation. In hindlimb ischaemia in the TSP-1-knockout mice, enhanced angiogenesis and improved blood flow were shown [26,27].

We were interested to find that when media from microvascular human endothelial cells cultured in hypoxia were added to proliferating endothelial cells, proliferation was blocked and apoptosis was induced. These effects were not observed when media from hypoxic muscle cells were used. This suggests the presence of a factor released by the hypoxic endothelial cells which was blocking proliferation. This effect was reduced by diluting the conditioning media, removing proteins that bind heparin and filtering large molecular weight proteins. This led us to consider whether TSP-1, a large glycoprotein with a heparin-binding domain, was a component of the culture media contributing to its antiproliferative and apoptotic effect. In keeping with this idea, we found that the concentration of TSP-1 was increased in media from hypoxic endothelial cells compared with normoxic endothelial cells and that TSP-1 mRNA levels were induced by hypoxic culture. Also, depletion of TSP-1 by immunoprecipitation partially blocked the inhibition of proliferation. These data are consistent with those in previous studies showing that TSP-1 can induce apoptosis of cells and that TSP-1 is hypoxia-inducible under some culture conditions [28,29]. Furthermore, when we directly treated proliferating endothelial cells with recombinant TSP-1, there was suppression of proliferation and induction of apoptosis.

We also have shown that there is more TSP-1 expressed in the dermis and epidermis of patients with SSc than in healthy controls, with staining in keeping with the presence of TSP-1 in keratinocytes and dermal connective tissue. These results are consistent with published data showing that TSP-1 is elevated in SSc [15]. Interestingly, the plasma levels of TSP-1 seen in SSc (mean ± SEM TSP-1 27.2 ± 8.5 ng/mL) are close to the levels that we found blocked endothelial cell proliferation and induced apoptosis in cultured endothelial cells.

One interesting result is that despite the induction of VEGF in hypoxia-conditioned media, which would be expected to support cell survival and proliferation, the increased levels of TSP-1 were able to override these protective effects. In support of this idea, TSP-1 has recently been shown to modulate the responses of endothelial cells to VEGF via interaction with the VEGF type 2 receptor [30].

TSP-1 has been studied previously in SSc and TSP-1 gene expression in skin correlates with severity of skin involvement in SSc and has been used in combination with other factors as a biomarker for the disease process [31]. In SSc and control fibroblasts, TSP-1 is induced by culture under hypoxic conditions [32]. In addition, TSP-1 activates the latent complex of transforming growth factor β (TGFβ) and maintains an autocrine loop of stimulation in SSc fibroblast by TGFβ [33]. It seems likely that TSP-1-dependent effects occur in the hypoxic microenvironment of SSc and that TSP-1 contributes to the microvascular injury and failure of repair and/or angiogenesis, despite the elevation of VEGF in the disease.

## Conclusions

Taken together, our data are consistent with a model wherein injured microvascular cells in SSc fail to repair because of dysregulated induction of TSP-1 in the hypoxic tissues. Antiangiogenic factors can be targeted in future therapeutic approaches, both in SSc and in peripheral vascular disease, where TSP-1 is also induced and blocks microvascular repair [34].

## Abbreviations

ABC: avidin-biotin complex; BSA: bovine serum albumin; DMEM: Dulbecco's modified Eagle's medium; ELISA: enzyme-linked immunosorbent assay; FCS: foetal calf serum; HMEC-1: human microvascular endothelial cell 1; PBS: phosphate-buffered saline; qRT-PCR: quantitative real-time polymerase chain reaction; SSc: systemic sclerosis; SV40: simian virus 40; TSP-1: thrombospondin 1; VEGF: vascular endothelial growth factor.

## Competing interests

The authors declare that they have no competing interests.

## Authors' contributions

LMR collected patient specimens and wrote the body of the article. JN performed the immunohistochemistry and qPCR analysis. XS performed cell culture and Western blot analysis. AL provided advice regarding matricellular protein biology and oversaw the cell biology experiments. JT provided specialist vascular biology advice and assisted in HMEC-1 culture. DA provided the laboratory facilities and an overview of the experimental protocols and results. RS conceived the study, performed tissue culture experiments and contributed to the writing of the article. All authors read and approved the final manuscript.
